# Ultra-low dose chest CT with silver filter and deep learning reconstruction significantly reduces radiation dose and retains quantitative information in the investigation and monitoring of lymphangioleiomyomatosis (LAM)

**DOI:** 10.1007/s00330-024-10649-z

**Published:** 2024-02-22

**Authors:** Alexa E. Golbus, Chloe Steveson, John L. Schuzer, Shirley F. Rollison, Tat’Yana Worthy, Amanda M. Jones, Patricia Julien-Williams, Joel Moss, Marcus Y. Chen

**Affiliations:** 1 Cardiovascular Branch, National Heart, Lung, and Blood Institute, National Institutes of Health, 10 Center Dr, MSC 1046, Building 10, Room B1D47, Bethesda, MD 20892 USA; 2 Canon Medical Systems, Otawara, Japan; 3 Radiology and Imaging Sciences, Clinical Center, National Institutes of Health, Bethesda, USA; 4 Office of the Clinical Director, National Heart, Lung, and Blood Institute, National Institutes of Health, Bethesda, USA; 5 Critical Care Medicine and Pulmonary Branch, National Heart, Lung, and Blood Institute, National Institutes of Health, Bethesda, USA

**Keywords:** Deep learning, Radiation dosage, Tomography (X-ray computed), Lung diseases

## Abstract

**Purpose:**

Frequent CT scans to quantify lung involvement in cystic lung disease increases radiation exposure. Beam shaping energy filters can optimize imaging properties at lower radiation dosages. The aim of this study is to investigate whether use of SilverBeam filter and deep learning reconstruction algorithm allows for reduced radiation dose chest CT scanning in patients with lymphangioleiomyomatosis (LAM).

**Material and methods:**

In a single-center prospective study, 60 consecutive patients with LAM underwent chest CT at standard and ultra-low radiation doses. Standard dose scan was performed with standard copper filter and ultra-low dose scan was performed with SilverBeam filter. Scans were reconstructed using a soft tissue kernel with deep learning reconstruction (AiCE) technique and using a soft tissue kernel with hybrid iterative reconstruction (AIDR3D). Cyst scores were quantified by semi-automated software. Signal-to-noise ratio (SNR) was calculated for each reconstruction. Data were analyzed by linear correlation, paired *t*-test, and Bland–Altman plots.

**Results:**

Patients averaged 49.4 years and 100% were female with mean BMI 26.6 ± 6.1 kg/m^2^. Cyst score measured by AiCE reconstruction with SilverBeam filter correlated well with that of AIDR3D reconstruction with standard filter, with a 1.5% difference, and allowed for an 85.5% median radiation dosage reduction (0.33 mSv vs. 2.27 mSv, respectively, *p* < 0.001). Compared to standard filter with AIDR3D, SNR for SilverBeam AiCE images was slightly lower (3.2 vs. 3.1, respectively, *p* = 0.005).

**Conclusion:**

SilverBeam filter with deep learning reconstruction reduces radiation dosage of chest CT, while maintaining accuracy of cyst quantification as well as image quality in cystic lung disease.

**Clinical relevance statement:**

Radiation dosage from chest CT can be significantly reduced without sacrificing image quality by using silver filter in combination with a deep learning reconstructive algorithm.

**Key Points:**

• *Deep learning reconstruction in chest CT had no significant effect on cyst quantification when compared to conventional hybrid iterative reconstruction.*

• *SilverBeam filter reduced radiation dosage by 85.5% compared to standard dose chest CT.*

• *SilverBeam filter in coordination with deep learning reconstruction maintained image quality and diagnostic accuracy for cyst quantification when compared to standard dose CT with hybrid iterative reconstruction.*

**Graphical abstract:**

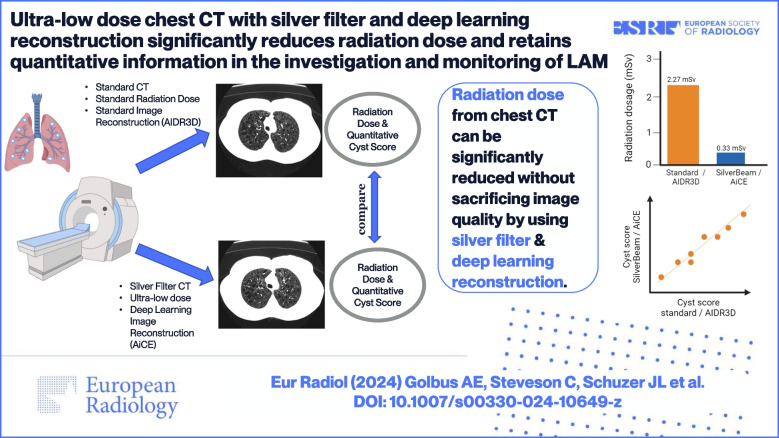

## Introduction

Lymphangioleiomyomatosis (LAM) is a neoplastic disease, predominantly affecting women, and is characterized by infiltrative abnormal smooth muscle–like cells causing diffuse thin-walled pulmonary cysts [1]. While histologically benign, LAM cells express the lymphangiogenic growth factors VEGF-C and VEGF-D and spread through lymphatic channels [2]. Cystic destruction of the lungs leads to progressive deterioration of lung function [3]. The mTOR1 inhibitor sirolimus (rapamycin) is first-line treatment [4].

Quantification of lung involvement by CT scans can be performed [5]. The cyst score, defined as percentage of lung volume with lower attenuation and thus occupied by cysts, correlates to LAM disease severity as well as to physiologic data including pulmonary function testing and ventilation-perfusion scintigraphy [6, 7]. CT is superior to chest radiographs in evaluating extent of disease, particularly in identifying cysts < 1.0 cm in diameter. Disease progression and quantification of treatment response can be monitored by serial CT scans [7]. However, frequent CT scans increase radiation exposure. Ionizing radiation increases risk of hematologic and solid organ cancers [8–10]. Malignancy risk following exposure is increased in younger patients and in females [11, 12]. Therefore, methods to reduce radiation doses while maintaining accurate CT cyst score quantification is important in this population given need for regular CT scans.

Attempts to reduce CT radiation dosage have included the use of X-ray beam filtration. Tin and copper filters reduce radiation dosage without negatively impacting image quality, with copper filters now being the standard. However, silver filters have recently been studied for dose reduction [13, 14]. Although recent studies have assessed the impact of silver filter in combination with deep learning reconstruction on image quality and radiation doses in phantom chest models, this has not been studied in clinical practice [8, 13, 15]. Image noise in ultra-low dose CT significantly degrades image quality when reconstructed with conventional methods; however, deep learning reconstruction can address this [16]. Deep learning reconstruction algorithms are known to improve image quality as assessed by signal-to-noise ratio (SNR) and contrast-to-noise ratio and have faster reconstruction speeds, compared to other reconstructions [8].

The aim of this study is to investigate whether the use of SilverBeam filter and a deep learning reconstruction algorithm allows for reduced radiation dose chest CT scanning.

## Methods

With institutional review board approval (96-H-0100) and informed consent, 60 consecutive patients with lymphangioleiomyomatosis (LAM) were enrolled in this prospective study. Patients underwent chest CT at standard and ultra-low radiation doses on a 320-detector row CT scanner (Aquilion One Prism Edition, Canon Medical Systems) at a single center from November 2021 through August 2022. All CT scans were performed with 80 × 0.5 mm helical acquisition mode with 0.5 s gantry rotation time and standard pitch. For standard dose CT scans, X-ray tube potential and current were determined by automatic exposure control (SUREExposure 3D, Canon Medical), based on scout image attenuation, and used an image quality factor of SD12.5 and reconstructed using a soft tissue kernel with hybrid iterative reconstruction (Adaptive Iterative Dose Reduction (AIDR3D). For standard filter images, a standard copper filter was used. Low-dose CT scans applied a SilverBeam filter and used fixed 120 kV and 80 mA and reconstructed using a soft tissue kernel with deep learning reconstruction (Advanced intelligent Clear-IQ Engine (AiCE)) techniques. Scanner-reported radiation dose parameters were documented based on the dose-length-product (DLP), for both the standard and low-dose scan protocols. The effective radiation dose in millisieverts (mSv) was calculated by multiplying dose-length-product by a conversion coefficient of 0.014 (mSv·mGy-1·cm-1) [17].

**Fig. 1:**
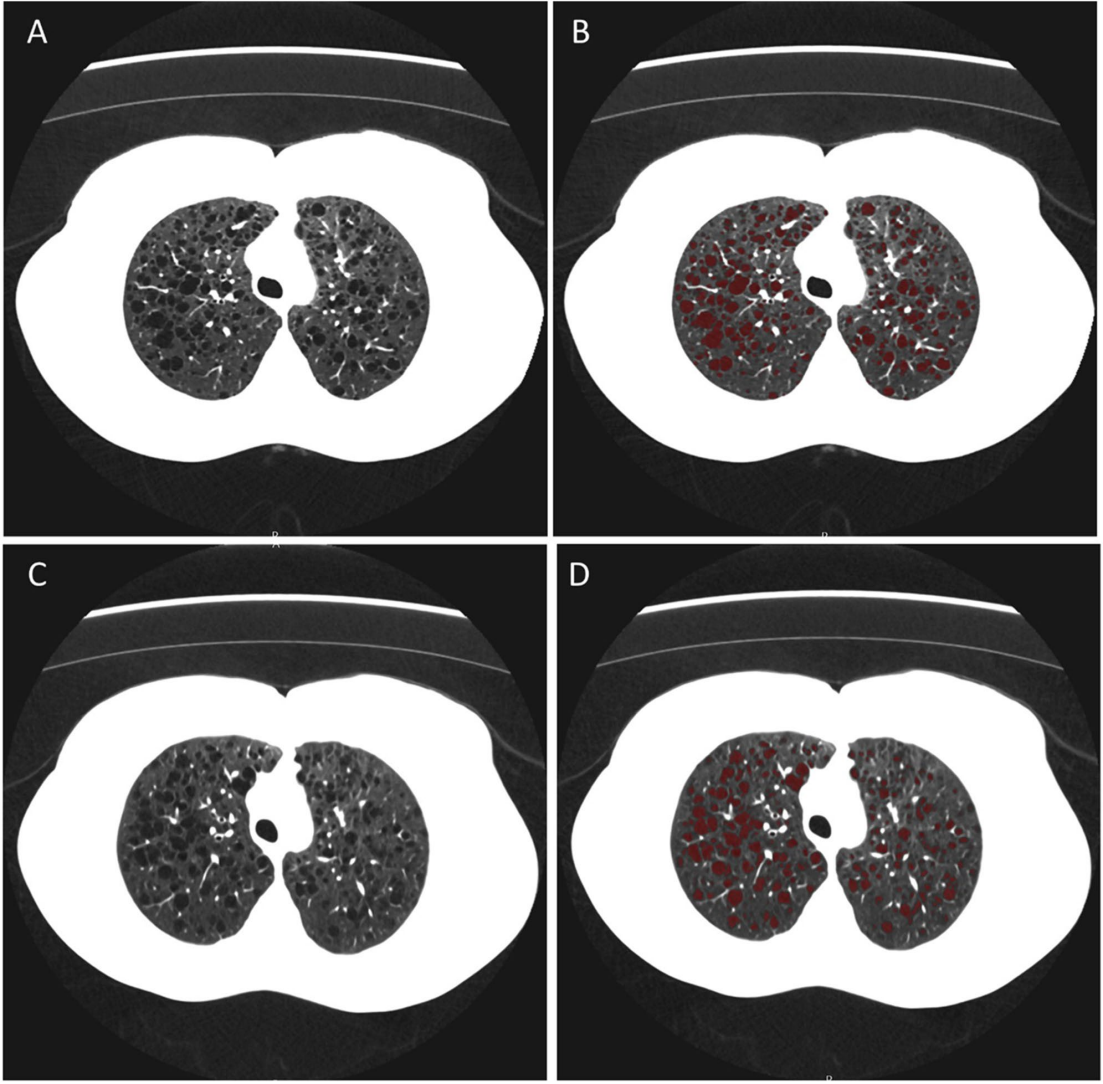
Chest CT of a 29-year-old female with LAM. **A** Standard scan with AIDR3D. **B** Standard scan with low attenuation areas highlighted in red. Radiation dose 1.90 mSv and cyst score 16.8%. **C** SilverBeam scan with AiCE. **D** SilverBeam scan with low attenuation areas highlighted in red. Radiation dose 0.33 mSv and cyst score 15.9%

Images were reconstructed with 512 × 512 matrix, 2 mm slice thickness with 1 mm slice interval. Cyst scores (% of lung volume affected by cysts) were quantified by semi-automated software (Lung Density Analysis, Canon Medical) (Fig. 1). Signal-to-noise ratio (SNR) was calculated for each reconstruction. Data was analyzed by linear correlation, paired *t*-test, and Bland–Altman plots.

## Results

Patients (*n* = 60) averaged 49.4 years (range 23–70 years) and 100% were female with mean BMI 26.6 ± 6.1 kg/m^2^ (Table 1).

**Table 1:** Demographics of 60 patients with LAM

Sample size (***n***)	60
Gender (%F)	100%
Age (years) (mean ± standard deviation)	49.4 ± 12.4
Height (cm) (mean ± standard deviation)	164.2 ± 7.2
Weight (kg) (mean ± standard deviation)	71.8 ± 17.2
BMI (mean ± standard deviation)	26.6 ± 6.1
Ethnicity	75% non-Hispanic White
	12% Asian
	5% Black
	5% Hispanic White
	3% unknown

**Fig. 2:**
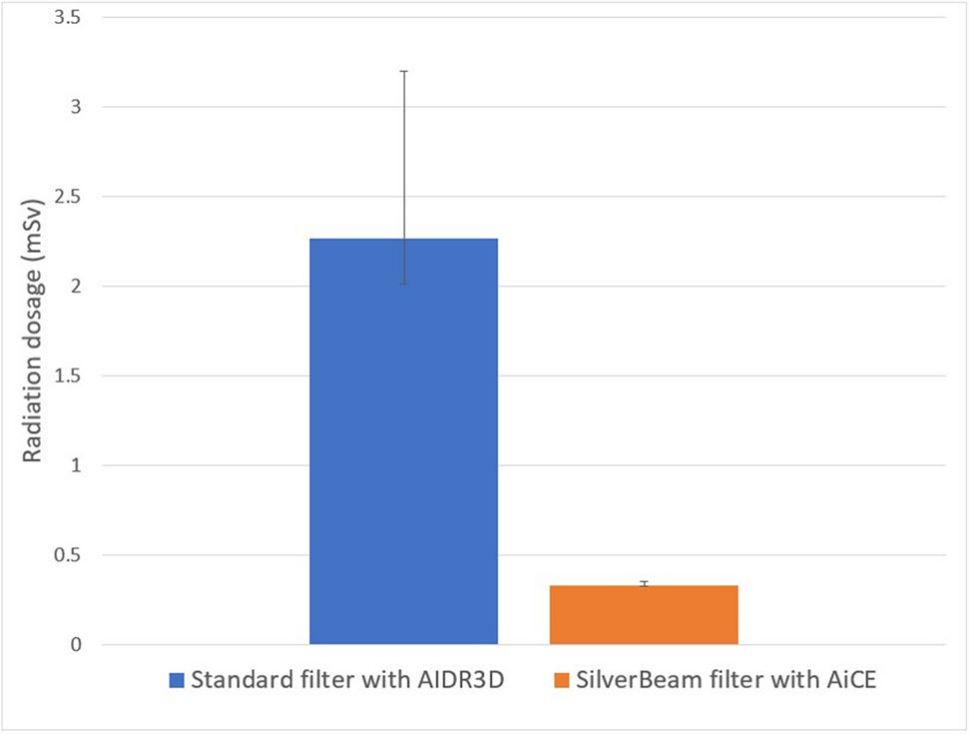
Median effective radiation dosage (mSv) of standard filter with AIDR3D versus SilverBeam filter with AiCE was 2.27 (IQR 2.01–3.20) vs. 0.33 (IQR 0.33–0.35) mSv, respectively (*p* < 0.001), representing an 85.5% radiation dosage reduction

**Fig. 3:**
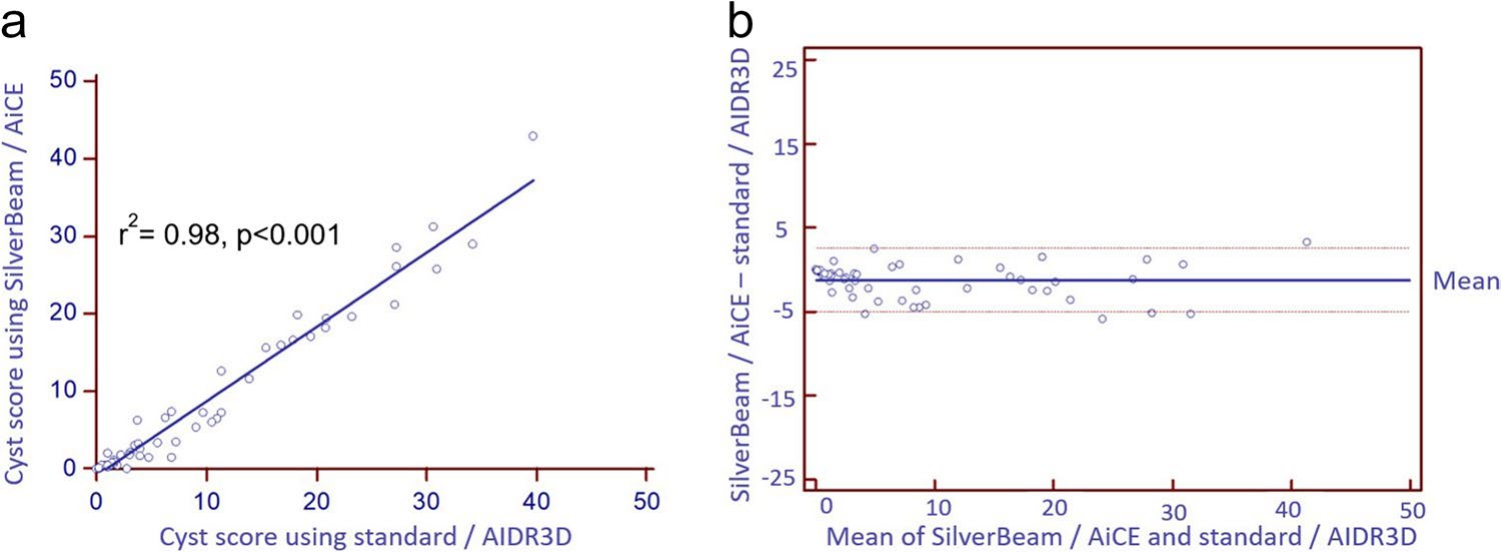
**a** Linear regression of cyst score using SilverBeam filter with AiCE reconstruction versus standard filter with AIDR3D reconstruction. Median cyst score for SilverBeam with AiCE was 2.4 (IQR 0.4–13.4)% versus 3.9 (IQR 1.0–14.3)% for standard filter with AIDR3D, *r*^2^ = 0.98, *p* < 0.001. **b** Bland–Altman plot showing mean negative bias of 1.23, with solid blue line indicating mean and red dashed lines indicating the bounds of the 95% confidence interval

**Fig. 4:**
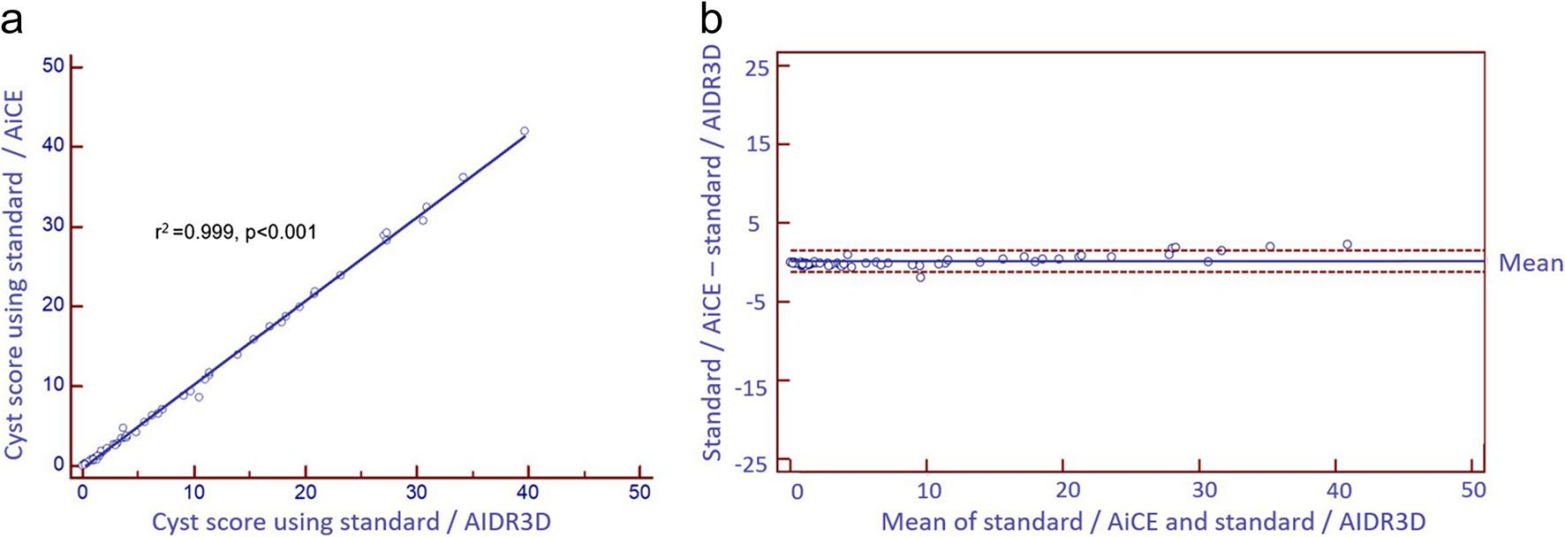
**a** Linear regression of cyst score for AiCE vs. AIDR3D reconstruction using standard filter. Median cyst score 3.7 (IQR 0.8–14.4)% vs. 3.9 (IQR 1.0–14.3)%, respectively, *r*^2^ = 0.99, *p* < 0.001. **b** Bland–Altman plot showing mean positive bias of 0.12 with solid blue line indicating mean and red dashed lines indicating the bounds of the 95% confidence interval

Ultra-low dose median radiation was reduced by 85.5% using SilverBeam filter with AiCE reconstruction compared to standard filter with AIDR3D reconstruction (0.33 (IQR 0.33–0.35) mSv vs. 2.27 (IQR 2.01–3.20) mSv, respectively, *p* < 0.001) (Fig. 2). The median cyst score was 2.4 (IQR 0.4–13.4)% for SilverBeam filter with AiCE reconstruction versus 3.9 (IQR 1.0–14.3)% for standard filter with AIDR3D reconstruction, representing a difference of 1.5%. Linear correlation coefficient for cyst score quantified with imaging using SilverBeam filter with AiCE versus standard filter with AIDR3D was excellent at 0.98 (*p* < 0.001) (Fig. 3a). Bland–Altman plot comparing difference in cyst score between the two showed a minimal negative bias of 1.23 (Fig. 3b). Linear correlation coefficient for cyst score of images reconstructed using AiCE compared to AIDR3D, with both using standard filter, was also excellent at 0.999 (*p* < 0.001, Fig. 4a), with median cyst score of 3.7 (IQR 0.8–14.4)% vs. 3.9 (IQR 1.0–14.3)%, respectively (*p* < 0.001). Bland–Altman plot showed minimal positive bias of 0.12 (Fig. 4b). Additionally, the linear correlation coefficient for cyst score quantified using SilverBeam filter with AIDR3D versus SilverBeam filter with AiCE was 0.98 (*p* < 0.001), while the linear correlation coefficient for cyst score quantified with using SilverBeam filter with AIDR3D versus standard filter with AIDR3D was 0.92 (*p* < 0.001).

Median signal-to-noise for imaging using SilverBeam filter with AiCE reconstruction was slightly lower than that of imaging using standard filter with AIDR3D reconstruction (3.1 (IQR 2.6–4.2) vs. 3.2 (IQR 2.7–4.7), respectively, *p* = 0.005). Median SNR for standard filter with AiCE was significantly greater than that of standard filter with AIDR3D (3.9 (IQR 3.3–5.5) vs. 3.2 (IQR 2.7–4.7), respectively, *p* < 0.001). SilverBeam filter with AiCE also had significantly greater SNR when compared to SilverBeam filter with AIDR3D (3.1 (IQR 2.6–4.2) vs. 1.9 (IQR 1.5–2.4), respectively, *p* < 0.001).

## Discussion

In our study, we evaluated the efficacy of SilverBeam filter with a deep learning reconstruction algorithm (AiCE) and found an 85.5% radiation dose reduction in chest CT scans of LAM patients. As pulmonary cysts in LAM are easily quantifiable, we utilized cyst score quantification as a metric to assess diagnostic accuracy of SilverBeam filter with AiCE. We compared the cyst score obtained using this filter and reconstruction to that quantified using the current traditional method and found that cyst score correlated strongly between images constructed using SilverBeam filter and AiCE reconstruction to those using standard filter and AIDR3D reconstruction, indicating that our method did not adversely impact diagnostic accuracy of cyst score quantification. Additionally, we demonstrated that the reconstructive method (AiCE) itself did not alter diagnostic accuracy, as the cyst score obtained using standard filter with AiCE reconstruction correlated very strongly to that obtained using this same filter with AIDR3D reconstruction. Nor did the SilverBeam filter itself negatively impact diagnostic accuracy, as there was a strong correlation in cyst score quantification between the SilverBeam filter and standard filter, with both using the same reconstructive method. Although signal-to-noise ratio was slightly lower for imaging using SilverBeam filter with AiCE reconstruction compared to that using standard filter with AIDR3D reconstruction, this difference of 0.1 is likely not large enough to be clinically significant. Comparing scans performed with the same filter but different reconstructions, AiCE allowed for a significantly greater SNR compared to AIDR3D, and can be used in conjunction with SilverBeam filter to improve image quality. Our results as a whole demonstrate that SilverBeam filter with AiCE reconstruction significantly reduces radiation dosage, while allowing for accurate cyst score quantification, without considerably affecting signal-to-noise ratio.

Although controversy exists surrounding the exact calculation of radiation risk, there have been recent advances in CT radiation dosage reduction [10], with one study showing that protocols including improved detector capability and tin filtration could reduce the risk of de novo lung cancer from lung cancer screening chest CT from 8.6 to 0.35 per 100,000 cases [10, 18]. In another study, standard dose chest CT (5 mSv) was shown to cause an increase in unstable chromosomal aberrations including DNA double strand breaks, whereas low-dose CT (classified as 1.5 mSv) did not [19]. In our study, SilverBeam filter reduced radiation to 0.33 mSv, which is below the threshold level of 1.5 mSv observed in this prior study. While radiation dose reduction in patients with LAM has previously been undertaken with ultra-low dose chest CT in combination with model based iterative reconstruction (MBIR), with a resulting 96% reduction in radiation (to a dosage of 0.14 mSv) [20], MBIR has lengthy computational times and image texture has “plastic” or “artificial” appearance [8]. In our study, we were able to significantly reduce radiation dose, although not nearly to the extent of this prior study [20]; however, we were able to avoid sacrificing image quality by using deep learning image reconstruction (AiCE) rather than MBIR.

Silver X-ray beam spectral modification filters have recently been studied for radiation dose reduction [13–15]. Nomura et al. [14] evaluated the ability of a silver filter to reduce radiation of localizer radiography and found a 74% reduction in radiation dosage, compared to localizer radiographs using a copper filter. This study found that although detectability of anatomical landmarks was not negatively impacted, the image noise was significantly greater with silver filter compared to copper filter. While this study only evaluated radiation reduction of a silver filter in localizer imaging, we evaluated radiation reduction using a silver filter for the CT scan itself and found a similar percentage reduction in radiation. However, we additionally utilized deep learning (AiCE) reconstruction to preserve image quality and reduce noise despite the reduction in radiation dosage, and were largely able to maintain image quality as assessed by signal-to-noise ratio. Kawamato et al. evaluated radiation dose characteristics and image quality when using silver filter with hybrid iterative reconstruction (AIDR3D) in phantom chests with a 10-mm nodule and found a reduction in image noise and artifacts when using a silver filter versus using no filter [15]. 

Recently, the ability of silver filter in combination with deep learning reconstruction, to detect lung nodules on phantom chests while reducing radiation dosage, was assessed [13]. Deep learning reconstruction (DLR) is a relatively new reconstructive method in CT that allows for preservation of image quality at lower radiation doses. While DLR has been evaluated in lung imaging including in nodule detection [21] and emphysema quantification [22], the combination of silver filters with DLR has only been evaluated in chest phantoms [13]. This study using phantom chests demonstrated that silver filter in combination with deep learning reconstruction reduces radiation dosage and increases median image quality score and nodule detection, compared to standard copper filter, with signal-to-noise ratio significantly greater for CT protocols using silver filter versus copper filter. We similarly found a reduction in radiation dosage using SilverBeam filter. However, we found SNR to be slightly lower with silver filter compared to standard copper filter. While this prior study had limitations specific to using phantom chests—particularly a lack of motion artifacts and no variability in body habitus that is present in clinical practice, as well as nodules that were perfectly round [13]—we demonstrated that this reduction in radiation dosage is maintained in real patients in clinical practice when using SilverBeam filter with deep learning reconstruction. Therefore, while these prior studies using silver filter [13–15] had similar findings in terms of radiation reduction, our study was the first to assess radiation reduction using silver filter in combination with deep learning reconstruction, in CT scans of real patients.

While our study evaluated detection of pulmonary cysts in patients with LAM, a rare disease, the cyst score in LAM is evaluated by measuring percentage of low attenuation volume on CT. Similar methods to quantify the extent of airway and tissue involvement can assess disease severity in other emphysematous diseases such as COPD, and has been shown to correspond to histological measurements [16, 23, 24]. A deep learning convolutional neural network has been studied in combination with ultra-low dose CT in emphysema quantification indices [22]. Emphysema indices of ultra-low dose scans reconstructed with DLR did not significantly differ from that of standard-dose CT scans reconstructed with an adaptive statistical iterative reconstruction. Further, image noise was not different between the two, despite a reduction in radiation dosage from 3.43 mSv for standard dose scans to 0.39 mSv for ultra-low dose scans [22]. However, silver filter to reduce radiation dosage has not been evaluated in emphysema alone or otherwise in combination with a DLR algorithm.

Although our study is a single-center study, we expect this technique will yield similar results at other centers. The lower radiation dose scan utilized a fixed technique without attenuation-based tube current modulation and therefore, image quality and radiation dose savings are proportional to body size. While the cysts in LAM are homogenous, well delineated, and easily quantifiable, we expect that SilverBeam filter with deep learning reconstruction in CT imaging in other diseases is likely to yield similar results in terms of radiation reduction with preservation of image quality and diagnostic accuracy. However, evaluation in other pulmonary diseases with less homogenous patterns is needed. Our findings suggest that SilverBeam filter in combination with deep learning reconstruction would likely be effective in reducing radiation dosage in imaging of other cystic or emphysematous diseases. SilverBeam filter with DLR allows for a significant reduction in radiation while maintaining image quality and diagnostic accuracy in cystic lung disease.

## Abbreviations

AiCE: Advanced intelligent Clear-IQ Engine
AIDR3D: Adaptive Iterative Dose Reduction
DLR: Deep learning reconstruction
LAM: Lymphangioleiomyomatosis
SNR: Signal-to-noise ratio

## Declarations

### Guarantor

The scientific guarantor of this publication is Marcus Chen.

### Statistics and biometry

No complex statistical methods were necessary for this paper.

### Study subjects or cohorts overlap

No study subjects or cohorts have been previously reported.

### Methodology

• prospective

• experimental

• performed at one institution
